# The Natural History of Small Vestibular Schwannomas

**DOI:** 10.7759/cureus.22231

**Published:** 2022-02-15

**Authors:** Serra L Aktan, Sarah Finucane, Matthew Kircher, Dennis Moore, Mariah Bashir

**Affiliations:** 1 Radiology, Loyola University Medical Center, Maywood, USA; 2 Otolaryngology - Head and Neck Surgery, Loyola University Medical Center, Maywood, USA

**Keywords:** mri - magnetic resonance imaging, neuroradiology, internal auditory canal lesion, vestibular schwannoma, accoustic neuroma

## Abstract

Objective

The incidence of vestibular schwannomas is increasing, and the average tumor size at diagnosis is decreasing. Therefore, understanding the specific growth pattern of small vestibular schwannomas is becoming increasingly important to guide clinical management. The objectives of this study were to evaluate the growth patterns of very small intracanalicular vestibular schwannomas measuring ≤ 4 mm in linear diameter and to assess the likelihood of these lesions ever requiring treatment.

Methods

A retrospective review was performed. A search of all MRI brain and internal auditory canal studies suggestive of a vestibular schwannoma from 1995 to 2019 was performed at our institution. This resulted in 372 cases, which were then evaluated for the presence of a vestibular schwannoma measuring ≤ 4 mm. All patients had to have at least one follow-up MRI to be included. Images were reviewed by a neuroradiologist.

Results

Eight ≤ 4 mm vestibular schwannomas were found that met all search criteria. The distribution of tumor sizes was as follows: three 2 mm, one 3 mm and four 4 mm. None of the ≤ 4 mm vestibular schwannomas identified demonstrated any significant growth in the linear dimension defined as greater than 2 mm of growth over observation times of 1-13 years (mean 6.3 years). None of the lesions ever required a treatment intervention per available medical records.

Conclusion

None of the ≤ 4 mm intracanalicular vestibular schwannomas identified in this study grew significantly or required treatment. Overall, the findings in this study suggest that vestibular schwannomas measuring ≤ 4 mm are unlikely to grow and ever require treatment.

## Introduction

This research was previously presented in virtual poster format at the American Society of Neuroradiology Annual Meeting May 30-June 4, 2020.

Vestibular schwannoma (VS) is a slow-growing, benign neoplasm of the eighth cranial nerve [1]. The most common symptoms associated with VS are asymmetric sensorineural hearing loss, asymmetric/unilateral subjective tinnitus and sudden sensorineural hearing loss [2,3]. Early diagnosis of these lesions is important for the preservation of hearing, facial nerve, and neurologic function. The most sensitive audiologic criteria that indicate the need for further evaluation of a possible VS include ≥10 decibels (dB) of interaural difference at 2 or more contiguous frequencies or ≥15 dB at a single frequency [3]. However, the most specific audiologic screening method with the highest positive predictive value for VS is ≥15 dB of interaural difference at 3,000 Hz [3]. The gold standard for diagnosing a VS is contrast-enhanced magnetic resonance imaging (MRI) with dedicated skull base imaging.

Growth rates and patterns for VSs have been previously studied; however, the growth pattern specifically of small VSs measuring less than 5 mm is not well-understood. As more MRI brain exams are done, more incidental lesions are being discovered, resulting in an increased incidence of VSs and decreased average tumor size at diagnosis [4]. Per Marinelli et al. [4], the average intracanalicular VS size at diagnosis has decreased from a median of 0.8 cm to 0.5 cm to 0.4 cm over the past three decades. Therefore, knowledge of the growth patterns of these small tumors is becoming increasingly important to guide treatment decisions. The specific aim of the current study is to investigate the growth rate of VSs measuring ≤ 4 mm and to estimate the likelihood of these very small tumors ever requiring a treatment intervention.

## Materials and methods

Study design

This study was approved by the Loyola Institutional Review Board (IRB) with IRB number 212345. A retrospective review was performed in order to evaluate the growth rate of very small VSs. Loyola University Medical Center’s radiologic and pathologic information system, Illuminate Insight (Illuminate Insight, Version 4.0, Softek, Overland Park KS), was used to search all the contrast-enhanced MRI of the brain and internal auditory canal (IAC) studies from 1995 to 2019. Search criteria were chosen collectively by the authors with the intention of conducting a broad search to find as many < 5 mm untreated VSs as possible. The search criteria are listed in Table 1.

**Table 1:** Illuminate insight search criteria

Including the exact phrase:	Internal auditory
With at least one of the words:	Punctate, focus, foci, nodule, nodular, tiny
Without the words:	Postoperative, post op, residual, recurrent, residual/recurrent, scarring, granulation, outpouching, compression

A list of 372 MRI studies with reports indicating a possible VS was generated from the Illuminate Insight search. These studies were then individually evaluated for the presence of a VS measuring less than 5 mm on a baseline exam and the presence of a follow-up MR brain or IAC exam. VSs were measured in at least two planes for the largest linear dimension. The largest linear dimension on the initial and follow-up MRIs was recorded. Images were reviewed by a board-certified neuroradiologist with six years of experience. The electronic medical records of patients were reviewed for any surgical or nonsurgical interventions for their VS, as well as any presenting symptoms of their VS.

Inclusion and exclusion criteria

To be included in the study, patients had to have an untreated VS measuring less than 5 mm on initial MRI imaging. Patients also had to have a follow-up MRI of the untreated VS at least one year later in order to evaluate the change in VS size over time. If there were more than one follow-up MRI, the chronologically latest MRI of the untreated VS was used as the follow-up exam.

**Figure 1:**
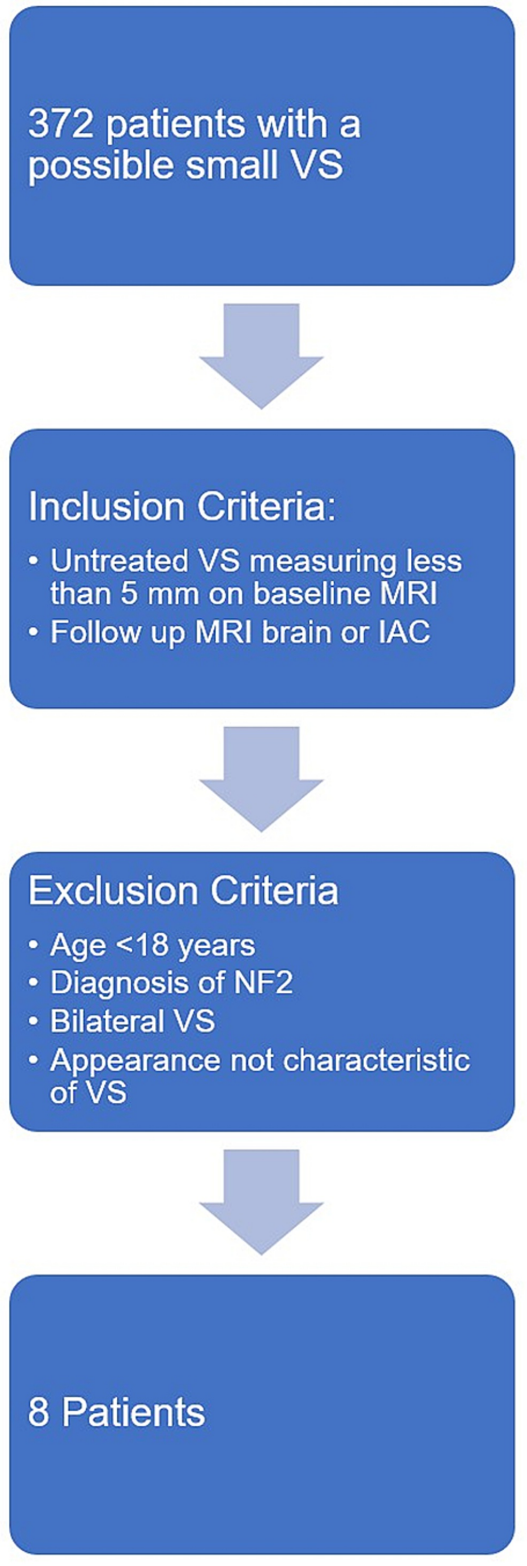
Patient selection process VS - Vestibular schwannoma; IAC - internal auditory canal; NF2 - neurofibromatosis 2

Any cases with imaging features not characteristic of a VS according to a board-certified neuroradiologist were excluded. Cases without a follow-up MRI were also excluded. Additional exclusion criteria were age <18 years, patients with bilateral VS or known neurofibromatosis 2 (NF2). An overview of the patient selection process is outlined in Figure 1.

## Results

**Figure 2:**
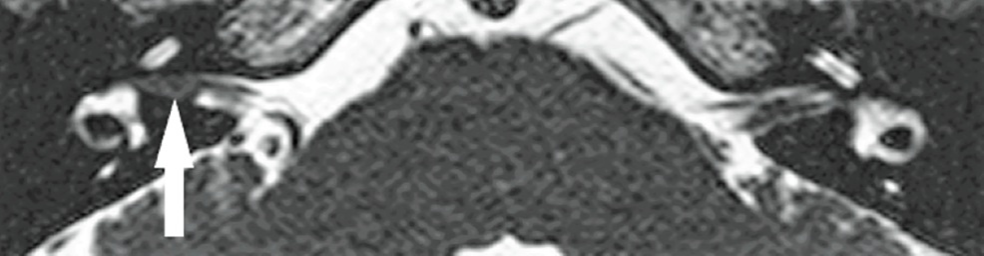
Axial 3D fast imaging employing steady-state acquisition (FIESTA) magnetic resonance image demonstrating a filling defect (arrow) in the right internal auditory canal consistent with a very small vestibular schwannoma.

**Figure 3:**
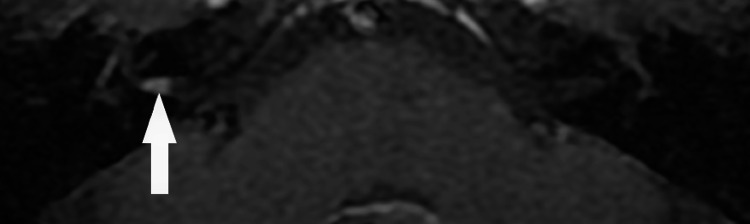
Axial T1 post-contrast magnetic resonance image with fat saturation of the same patient demonstrating enhancement (arrow) in the right internal auditory canal consistent with a very small vestibular schwannoma.

Our search resulted in eight patients with VS ≤4 mm in maximum linear dimension (Table 2) who met all inclusion criteria. We did not identify any intralabyrinthine schwannomas; all the VSs included in this study were intracanalicular. Representative MRI images of a very small VS from one of these eight patients is demonstrated in Figures 2, 3. Age at diagnosis ranged from 50 to 78 (mean 60.4 years, median 61 years). Four of the tumors were on the right, four on the left. The initial size ranged from 2-4 mm (mean 3.1). The distribution of VS sizes was as follows: three 2 mm, one 3 mm and four 4 mm. Four of the tumors were incidental findings, one was found in the evaluation of ipsilateral hearing loss, one for ipsilateral hearing loss with vertigo, one for vertigo alone and one for ipsilateral tinnitus. None of the VSs identified demonstrated any significant growth in the linear dimension defined as greater than 2 mm of growth [5] over observation times of 1-13 years (mean 6.3 years). Three VSs were found to have decreased in size over the observation period of 1, 5 and 12 years. None of the eight VSs ever required treatment intervention according to the available medical records. Two patients were excluded from the study because of a diagnosis of NF2.

**Table 2:** Patients with ≤4 mm vestibular schwannomas L = Left; R = Right; F = Female; M = Male *stable per reports for 13 years (initial images from 2,000 are unavailable), stable per available imaging for one year

Patient	Age	Sex	Side	Presentation	Duration (years)	Date 1	Date 2	Size 1 (mm)	Size 2 (mm)	Growth (mm)
1	62	F	R	Incidental	5	2006	2011	4	3	-1
2	50	F	L	L tinnitus	4	2006	2010	2	2	0
3	60	F	L	Incidental	5	2013	2018	4	5	+1
4	51	F	L	L hearing loss and vertigo	13	2000*	2013	2	2	0
5	55	F	L	Vertigo	1	2009	2010	4	2	-2
6	62	F	R	Incidental	2	2008	2010	2	2	0
7	78	M	R	R hearing loss	12	2007	2019	4	2	-2
8	65	M	R	Incidental	8	2006	2014	3	4	+1

## Discussion

Prior studies have investigated the growth rates of VSs, but not specifically those of very small VSs. In a 2005 systematic review, Yoshimoto et al. [6] determined that the mean growth rate for VSs is 1.2 mm/year (0.4 to 2.9 mm/yr) and that 18% required intervention during follow-up. In a large single-institution experience, Hunter et al. [7] concluded that larger tumor diameter at diagnosis was predictive of growth, with every centimeter increase in tumor dimension at diagnosis associated with a two-fold increase in the likelihood of growth. Lees et al. [8] studied the natural history of tumor growth in 361 sporadic VSs with a median follow-up of 4.1 years, of which 232 were intracanalicular at initial presentation. Lees et al. [8] stated “we found that larger tumors are more likely to demonstrate growth during conservative observation.” Agrawal et al. [9] also found that tumor size at diagnosis is predictive of growth, and they determined that the increased risk of tumor growth was 20% for each 1 mm increase in initial tumor size. Similarly, Eljamel et al. [10] found that tumor volume >1.2 cubic cm at presentation was a predictor of future growth (p=0.02). Given the results of these studies, one would expect the smallest VSs to have the lowest tendency for growth. This is directly supported by the findings in this study, where the natural history of our series of very small VS demonstrates no significant growth requiring intervention even over considerable follow-up time periods.

Furthermore, multiple studies have shown VSs are most likely to grow within the first several years after diagnosis. Lees et al. [8] found that only 4% of their 249 VS patients who experienced volumetric growth had growth first detected at > 5 years. Based on a large database of patients with a VS, Kirchmann et al. [11] showed that VSs are most likely to grow in the first few years after diagnosis, with few growing after 4.6 years of observation. Even excluding our cases in which there have been less than 4.6 years of follow-up (cases 2, 5 and 6), the remaining 5 study tumors had 5-13 years of follow-up (mean 7.4 years) and still demonstrated no growth. Half of the tumors were incidental findings, whereas symptomatic diagnosis has been found to be predictive of tumor growth [6]. It is possible that a larger cohort would have included at least some cases with significant tumor growth. As observed by Stangerup et al. [12], “only a limited number of VSs grow continuously; others do not, and some even shrink.”

In the future, a larger cohort of patients studied similarly may have implications for VS MRI screening protocols. The gold standard for VS diagnosis is a contrast-enhanced MRI of the internal auditory canals. However, non-contrast fast screening protocols have been developed to reduce cost, contrast reactions, and patient scanning time. Multiple prior studies investigating screening MRI protocols for VS have reported high accuracy, but false negatives primarily occur when tumors measure ≤ 4 mm [5,13,14]. For example, Abele et al. [13] studied a screening protocol using unenhanced axial T2 3D CISS (constructive interference in steady-state) and coronal T2 sequences and reported two false negatives involving a 3 mm and a 4 mm lesion. The results of our series preliminarily support the use of non-contrast screening MRI protocols since the very small tumors which are most likely to be undiagnosed on screening MRIs (false negative) would not be expected to grow or require a treatment intervention.

There are several limitations of this study. This search yielded only eight very small VSs, which is too small for meaningful statistical analysis. The need for a follow-up MRI reduced the number of studies we were able to include in the study. In addition, very small VSs, particularly when incidental, maybe under-reported by dictating radiologists. Measurements of the patient's hearing were not available to be included in this study. All patients in this study were 50 years or older, potentially underrepresenting younger patients. However, in a review by Paldor et al. [15], 20 of 22 studies evaluating age and VS growth showed no significant association between age and growth. The most commonly observed definition of linear VS growth during observational management is 2mm; clearly, this has little meaning for the very small VS. We utilized linear measurements only, not volumetric measurements. However, van de Langenberg et al. [16] compared linear vs volumetric measurement of VS and found that for smaller tumors the measurement error for both techniques was comparable. The VSs identified in this study were only followed for a mean of 5.5 years (median 5 years). Stangerup et al. [12] stated that “Regardless of tumor localization or size, growth only occurs within the first 5 years after the diagnosis” in their study of Natural History of Vestibular Schwannoma. Additionally, Lees et al. [8] found that only 4% of their 249 VS patients who experienced volumetric growth had growth first detected at > 5 years. Finally, this study was a retrospective review and subject to the inherent weaknesses of this study type.

## Conclusions

The results of this study suggest that the smallest intracanalicular VSs do not grow significantly and therefore have a very low likelihood of ever requiring treatment. These findings are concordant with the existing literature on this topic. Future larger studies are required to definitively answer the question of the long-term stability of very small VSs.

## References

[1] Neff BA, Welling DB, Akhmametyeva E, Chang LS (2006). The molecular biology of vestibular schwannomas: dissecting the pathogenic process at the molecular level. Otol Neurotol.

[2] Foley RW, Shirazi S, Maweni RM, Walsh K, McConn Walsh R, Javadpour M, Rawluk D (2017). Signs and symptoms of acoustic neuroma at initial presentation: an exploratory analysis. Cureus.

[3] Sweeney AD, Carlson ML, Shepard NT, McCracken DJ, Vivas EX, Neff BA, Olson JJ (2018). Congress of neurological surgeons systematic review and evidence-based guidelines on otologic and audiologic screening for patients with vestibular schwannomas. Neurosurgery.

[4] Marinelli JP, Lohse CM, Carlson ML (2018). Incidence of vestibular schwannoma over the past half-century: A population-based study of Olmsted County, Minnesota. Otolaryngol Head Neck Surg.

[5] Liudahl AA, Davis AB, Liudahl DS, Maley J, Policeni B, Hansen MR (2018). Diagnosis of small vestibular schwannomas using constructive interference steady state sequence. Laryngoscope.

[6] Yoshimoto Y (2005). Systematic review of the natural history of vestibular schwannoma. J Neurosurg.

[7] Hunter JB, Francis DO, O'Connell BP (2016). Single institutional experience with observing 564 vestibular schwannomas: factors associated with tumor growth. Otol Neurotol.

[8] Lees KA, Tombers NM, Link MJ (2018). Natural history of sporadic vestibular schwannoma: a volumetric study of tumor growth. Otolaryngol Head Neck Surg.

[9] Agrawal Y, Clark JH, Limb CJ, Niparko JK, Francis HW (2010). Predictors of vestibular schwannoma growth and clinical implications. Otol Neurotol.

[10] Eljamel S, Hussain M, Eljamel MS (2011). Should initial surveillance of vestibular schwannoma be abandoned?. Skull Base.

[11] Kirchmann M, Karnov K, Hansen S, Dethloff T, Stangerup SE, Caye-Thomasen P (2017). Ten-year follow-up on tumor growth and hearing in patients observed with an intracanalicular vestibular schwannoma. Neurosurgery.

[12] Stangerup SE, Caye-Thomasen P, Tos M, Thomsen J (2006). The natural history of vestibular schwannoma. Otol Neurotol.

[13] Abele TA, Besachio DA, Quigley EP, Gurgel RK, Shelton C, Harnsberger HR, Wiggins RH 3rd (2014). Diagnostic accuracy of screening MR imaging using unenhanced axial CISS and coronal T2WI for detection of small internal auditory canal lesions. AJNR Am J Neuroradiol.

[14] Allen RW, Harnsberger HR, Shelton C (1996). Low-cost high-resolution fast spin-echo MR of acoustic schwannoma: an alternative to enhanced conventional spin-echo MR?. AJNR Am J Neuroradiol.

[15] Paldor I, Chen AS, Kaye AH (2016). Growth rate of vestibular schwannoma. J Clin Neurosci.

[16] van de Langenberg R, de Bondt BJ, Nelemans PJ, Baumert BG, Stokroos RJ (2009). Follow-up assessment of vestibular schwannomas: volume quantification versus two-dimensional measurements. Neuroradiology.

